# Poly[aqua­(μ_3_-pyridazine-4-carboxyl­ato-κ^2^ *O*:*O*:*O*′)lithium]

**DOI:** 10.1107/S1600536811008634

**Published:** 2011-03-12

**Authors:** Wojciech Starosta, Janusz Leciejewicz

**Affiliations:** a Institute of Nuclear Chemistry and Technology, ul.Dorodna 16, 03-195 Warszawa, Poland

## Abstract

The structure of the title compound, [Li(C_5_H_3_N_2_O_2_)(H_2_O)]_*n*_, is composed of centrosymmetric dimers in which two Li^I^ ions are bridged by a carboxyl­ate O atom, each donated by a ligand, acting in a bidentate mode. The second carboxyl­ato O atoms bridge the dimers to Li^I^ ions in adjacent dimers, forming mol­ecular layers parallel to (001). Each Li^I^ ion is coordinated by two bridging carboxyl­ate O atoms, a bridging carboxyl­ate O atom donated by the adjacent dimer and an aqua O atom, resulting in a distorted tetra­hedral coordination geometry. The layers are held together by O—H⋯N hydrogen bonds in which coordinated water O atoms act as donors and ligand hetero-ring N atoms as acceptors.

## Related literature

For the crystal structure of a Pb(II) complex with pyridazine-4-carboxyl­ate and water ligands, see: Starosta & Leciejewicz, (2009 ▶) and for the structure of a Mg(II) complex, see: Starosta & Leciejewicz, (2011*b* ▶). For the structure of pyridazine-4-carb­oxy­lic acid hydro­chloride, see: Starosta & Leciejewicz, (2008 ▶) and for the structure of a Li^I^ complex with pyridazine-3-carboxyl­ate and water ligands, see: Starosta & Leciejewicz, (2011*a* ▶). 
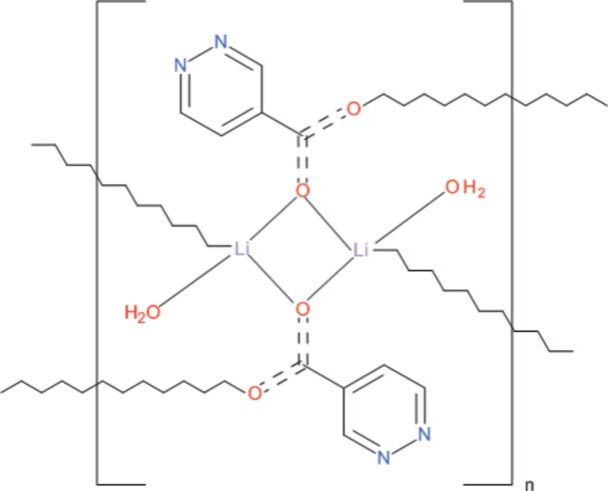


## Experimental

### 

#### Crystal data

[Li(C_5_H_3_N_2_O_2_)(H_2_O)]
*M* *_r_* = 148.05
Monoclinic, 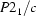

*a* = 8.1673 (16) Å
*b* = 9.6908 (19) Å
*c* = 8.0248 (16) Å
β = 97.08 (3)°
*V* = 630.3 (2) Å^3^
*Z* = 4
Mo *K*α radiation
μ = 0.13 mm^−1^
*T* = 293 K
0.30 × 0.28 × 0.12 mm

#### Data collection

Kuma KM-4 four-circle diffractometer
Absorption correction: analytical (*CrysAlis RED*; Oxford Diffraction, 2008) ▶ *T*_min_ = 0.946, *T*_max_ = 0.973
1958 measured reflections
1843 independent reflections
1208 reflections with *I* > 2σ(*I*)
*R*_int_ = 0.077
3 standard reflections every 200 reflections  intensity decay: 2.1%

#### Refinement

*R*[*F* ^2^ > 2σ(*F* ^2^)] = 0.039
*wR*(*F* ^2^) = 0.128
*S* = 1.02
1843 reflections
108 parameters
H atoms treated by a mixture of independent and constrained refinement
Δρ_max_ = 0.33 e Å^−3^
Δρ_min_ = −0.28 e Å^−3^

### 

Data collection: *KM-4 Software* (Kuma, 1996 ▶); cell refinement: *KM-4 Software*; data reduction: *DATAPROC* (Kuma, 2001 ▶); program(s) used to solve structure: *SHELXS97* (Sheldrick, 2008 ▶); program(s) used to refine structure: *SHELXL97* (Sheldrick, 2008 ▶); molecular graphics: *SHELXTL* (Sheldrick, 2008 ▶); software used to prepare material for publication: *SHELXTL*.

## Supplementary Material

Crystal structure: contains datablocks I, global. DOI: 10.1107/S1600536811008634/kp2313sup1.cif

Structure factors: contains datablocks I. DOI: 10.1107/S1600536811008634/kp2313Isup2.hkl

Additional supplementary materials:  crystallographic information; 3D view; checkCIF report

## Figures and Tables

**Table 1:** Selected bond lengths (Å)

O1—Li1	1.967 (2)
Li1—O2^i^	1.909 (3)
Li1—O3	1.915 (3)
Li1—O1^ii^	1.946 (2)

Symmetry codes: (i) 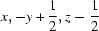; (ii) 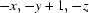.

**Table 2:** Hydrogen-bond geometry (Å, °)

*D*—H⋯*A*	*D*—H	H⋯*A*	*D*⋯*A*	*D*—H⋯*A*
O3—H32⋯N1^iii^	0.86 (3)	1.93 (3)	2.7910 (18)	175 (3)
O3—H31⋯N2^iv^	0.85 (3)	2.33 (3)	3.1272 (19)	155 (2)

Symmetry codes: (iii) 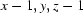; (iv) 
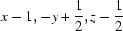
.

**Fig. 1.**
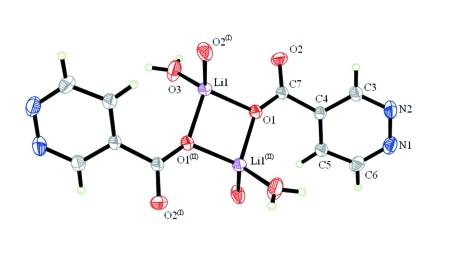
A dimeric structural unit of the title compound with atom labelling scheme and 50% probability displacement ellipsoids. Symmetry code: (I) x, -y + 1/2, z - 1/2. (II) -x, -y + 1, -z.

**Fig. 2.**
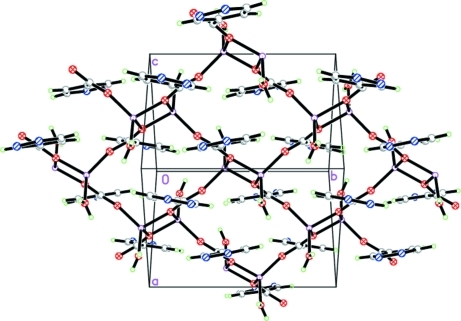
Molecular layer composed of dimeric structural units.

**Fig. 3.**
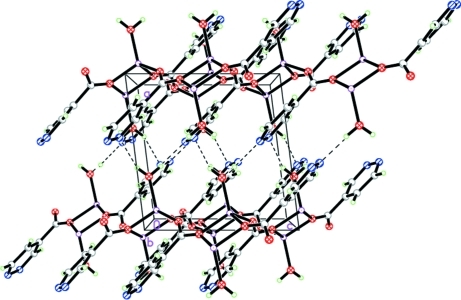
The packing of layers viewed along the c axis.

## supplementary crystallographic information

### Comment

The structural unit of the title compound is a centrosymmetric dimer composed of two Li^I^ ions bridged by a bidentate carboxylate O1 atom, each donated by one of two symmetry related ligands. The ligand carboxylate group C7/O1/O2 makes with the O1/Li1/O1^(II)^/Li1^(II)^ plane a dihedral angle of 10.9 (1)°, while the dihedral angle between this carboxylate group and the pyridazine ring is 43.4 (2)°. The pyridazine ring is almost planar with r.m.s. of 0.0148 (2) Å. Both ligand's heterocyclic N atoms remain coordination inactive. Bond distances and bond angles within the ligand molecule fit the values reported earlier in the structures of pyridazine-4-carboxylic acid hydrochloride (Starosta & Leciejewicz, 2008) and other metal complexes with the title ligand. The Li^I^ ion is coordinated by the bridging carboxylato O1 and O1^(II)^atoms, a bridging carboxylato O2^(I)^ atom donated by the adjacent dimer and the aqua O3 atom resulting in a distorted tetrahedral coordination. The observed Li—O bond distances which fall in the range from 1.909 (3) to 1.946 (2)Å are characteristic for all Li^I^ complexes with carboxylate ligands. Carboxylato O2 atoms bridge the dimers into molecular layers which are approximately parallel to the crystal *bc* plane. The structure of a layer can be visualized as composed of corrugated loops with four equal sides and the dimers at their apices. Hydrophobic parts of pairs of pyridazine rings are directed inside the loop with the closet distance of 4.91 (1)Å between the ring centers while the heterocyclic N atoms are directed outside and participate in a network of hydrogen bonds. The latter consists of coordinated water molecules acting as donors and the pyridazine N atoms in an adjacent layer as acceptors. They form centrosymmetric rings which give rise to a three-dimensional structure. Discrete dinuclear molecules were reported to constitute the structure of a Pb(II) complex with the title ligand, in which two symmetry related metal ions are bridged by a pair of ligands *via* their heterocyclic N atoms and two pairs of aqua O atoms. Each Pb(II) ion is also coordinated by two carboxylate O atoms of another ligand (Starosta & Leciejewicz, 2009). On the other hand, the structure of a Mg(II) complex is built of discrete centrosymmetric molecules in which the metal ion is coordinated by only one carboxylato O atom of two ligands and two pairs of aqua O atoms in octahedral geometry. Heterocyclic N atoms do not act in coordination mode (Starosta & Leciejewicz, 2011*b*). Discrete monomers have been also reported to constitute the structure of a Li^I^ complex with the pyridazine-3-carboxylate and water ligands. A Li^I^ ion is coordinated by ligand N,*O* chelating group and two aqua O atoms in a terahedral mode (Starosta & Leciejewicz, 2011*a*).

### Experimental

The title compound was obtained by boiling under reflux with stirring 50 ml of an aqueous solution containig 1 mmol of pyridazine-4-carboxylic acid (Aldrich) and 1 mmol of LiOH (Aldrich). The solution was boiled for two h. After cooling to room temperature a 1 N solution of HCl was added dropwise until the pH reached the value of 5.5 and then left to crystallize. Ten days later, colourless crystalline plates were found after evaporating to dryness. They were recrystallized from water a couple of times until well formed single crystals were found. They were washed with cold ethanol and dried in air.

### Refinement

Water hydrogen atoms were located in a difference map and refined isotropically. H atoms arrached to pyridazine-ring C atoms were positioned at calculated positions and were treated as riding on the parent atoms, with C—H=0.93 Å and *U*_iso_(H)=1.5*U*_eq_(C).

### Crystal data

**Table:**

[Li(C_5_H_3_N_2_O_2_)(H_2_O)]	*F*(000) = 304
*M**_r_* = 148.05	*D*_x_ = 1.560 Mg m^−^^3^
Monoclinic, *P*2_1_/*c*	Mo *K*α radiation, λ = 0.71073 Å
Hall symbol: -P 2ybc	Cell parameters from 25 reflections
*a* = 8.1673 (16) Å	θ = 6–15°
*b* = 9.6908 (19) Å	µ = 0.13 mm^−^^1^
*c* = 8.0248 (16) Å	*T* = 293 K
β = 97.08 (3)°	Plates, colourless
*V* = 630.3 (2) Å^3^	0.30 × 0.28 × 0.12 mm
*Z* = 4	

### Data collection

**Table:**

Kuma KM-4 four-circle diffractometer	1208 reflections with *I* > 2σ(*I*)
Radiation source: fine-focus sealed tube	*R*_int_ = 0.077
graphite	θ_max_ = 30.1°, θ_min_ = 2.5°
profile data from ω/2θ scans	*h* = 0→11
Absorption correction: analytical (*CrysAlis RED*; Oxford Diffraction, 2008)	*k* = −13→0
*T*_min_ = 0.946, *T*_max_ = 0.973	*l* = −11→11
1958 measured reflections	3 standard reflections every 200 reflections
1843 independent reflections	intensity decay: 2.1%

### Refinement

**Table:**

Refinement on *F*^2^	Primary atom site location: structure-invariant direct methods
Least-squares matrix: full	Secondary atom site location: difference Fourier map
*R*[*F*^2^ > 2σ(*F*^2^)] = 0.039	Hydrogen site location: inferred from neighbouring sites
*wR*(*F*^2^) = 0.128	H atoms treated by a mixture of independent and constrained refinement
*S* = 1.02	*w* = 1/[σ^2^(*F*_o_^2^) + (0.0832*P*)^2^ + 0.0472*P*] where *P* = (*F*_o_^2^ + 2*F*_c_^2^)/3
1843 reflections	(Δ/σ)_max_ < 0.001
108 parameters	Δρ_max_ = 0.33 e Å^−^^3^
0 restraints	Δρ_min_ = −0.28 e Å^−^^3^

### Special details

**Table:**

Geometry. All e.s.d.'s (except the e.s.d. in the dihedral angle between two l.s. planes) are estimated using the full covariance matrix. The cell e.s.d.'s are taken into account individually in the estimation of e.s.d.'s in distances, angles and torsion angles; correlations between e.s.d.'s in cell parameters are only used when they are defined by crystal symmetry. An approximate (isotropic) treatment of cell e.s.d.'s is used for estimating e.s.d.'s involving l.s. planes.
Refinement. Refinement of *F*^2^ against ALL reflections. The weighted *R*-factor *wR* and goodness of fit *S* are based on *F*^2^, conventional *R*-factors *R* are based on *F*, with *F* set to zero for negative *F*^2^. The threshold expression of *F*^2^ > σ(*F*^2^) is used only for calculating *R*-factors(gt) *etc*. and is not relevant to the choice of reflections for refinement. *R*-factors based on *F*^2^ are statistically about twice as large as those based on *F*, and *R*- factors based on ALL data will be even larger.

### Fractional atomic coordinates and isotropic or equivalent isotropic displacement parameters (Å^2^)

**Table:**

	*x*	*y*	*z*	*U*_iso_*/*U*_eq_	
O1	0.03194 (12)	0.42855 (10)	0.15091 (11)	0.0264 (2)	
C4	0.18403 (15)	0.37648 (13)	0.41306 (14)	0.0210 (2)	
O2	−0.03510 (14)	0.24156 (11)	0.28565 (13)	0.0369 (3)	
N2	0.40511 (15)	0.28845 (14)	0.60951 (16)	0.0334 (3)	
C7	0.04912 (15)	0.34645 (12)	0.27196 (15)	0.0210 (3)	
C6	0.33735 (19)	0.51936 (16)	0.61350 (17)	0.0316 (3)	
H6	0.3551	0.6058	0.6629	0.038*	
N1	0.42945 (15)	0.41548 (14)	0.67579 (15)	0.0334 (3)	
C3	0.28349 (16)	0.26953 (14)	0.48590 (16)	0.0263 (3)	
H3	0.2633	0.1804	0.4455	0.032*	
C5	0.21451 (17)	0.50626 (14)	0.47709 (16)	0.0262 (3)	
H5	0.1559	0.5825	0.4316	0.031*	
Li1	−0.1023 (3)	0.3958 (2)	−0.0663 (3)	0.0264 (5)	
O3	−0.33301 (14)	0.39117 (15)	−0.04432 (15)	0.0415 (3)	
H31	−0.389 (4)	0.354 (3)	0.027 (3)	0.075 (8)*	
H32	−0.403 (4)	0.403 (3)	−0.133 (4)	0.075 (8)*	

### Atomic displacement parameters (Å^2^)

**Table:**

	*U*^11^	*U*^22^	*U*^33^	*U*^12^	*U*^13^	*U*^23^
O1	0.0337 (5)	0.0224 (5)	0.0208 (4)	−0.0029 (3)	−0.0062 (3)	0.0050 (3)
C4	0.0245 (5)	0.0214 (5)	0.0163 (5)	−0.0005 (4)	−0.0008 (4)	0.0011 (4)
O2	0.0491 (6)	0.0269 (5)	0.0311 (5)	−0.0160 (4)	−0.0099 (4)	0.0068 (4)
N2	0.0301 (6)	0.0370 (7)	0.0308 (6)	0.0046 (5)	−0.0057 (4)	0.0065 (5)
C7	0.0259 (6)	0.0182 (6)	0.0178 (5)	0.0004 (4)	−0.0019 (4)	0.0000 (4)
C6	0.0357 (7)	0.0332 (7)	0.0241 (6)	−0.0053 (6)	−0.0035 (5)	−0.0046 (5)
N1	0.0302 (6)	0.0422 (7)	0.0255 (6)	−0.0032 (5)	−0.0057 (4)	0.0016 (5)
C3	0.0289 (6)	0.0249 (6)	0.0240 (6)	0.0022 (5)	−0.0009 (5)	0.0023 (5)
C5	0.0308 (6)	0.0231 (6)	0.0230 (6)	0.0007 (5)	−0.0036 (4)	−0.0006 (5)
Li1	0.0312 (11)	0.0179 (10)	0.0278 (11)	−0.0006 (8)	−0.0057 (8)	−0.0018 (8)
O3	0.0282 (5)	0.0601 (8)	0.0336 (6)	−0.0048 (5)	−0.0066 (4)	0.0073 (5)

### Geometric parameters (Å, °)

**Table:**

O1—C7	1.2501 (15)	C6—C5	1.3965 (18)
O1—Li1^i^	1.946 (2)	C6—H6	0.9300
O1—Li1	1.967 (2)	C3—H3	0.9300
C4—C5	1.3697 (18)	C5—H5	0.9300
C4—C3	1.3995 (17)	Li1—O2^iii^	1.909 (3)
C4—C7	1.5078 (17)	Li1—O3	1.915 (3)
O2—C7	1.2398 (16)	Li1—O1^i^	1.946 (2)
O2—Li1^ii^	1.909 (3)	Li1—Li1^i^	2.751 (4)
N2—C3	1.3272 (18)	O3—H31	0.85 (3)
N2—N1	1.3460 (19)	O3—H32	0.86 (3)
C6—N1	1.318 (2)		
			
C7—O1—Li1^i^	144.92 (11)	C4—C3—H3	118.2
C7—O1—Li1	125.64 (11)	C4—C5—C6	117.21 (12)
Li1^i^—O1—Li1	89.33 (10)	C4—C5—H5	121.4
C5—C4—C3	117.00 (11)	C6—C5—H5	121.4
C5—C4—C7	122.76 (11)	O2^iii^—Li1—O3	113.75 (12)
C3—C4—C7	120.23 (11)	O2^iii^—Li1—O1^i^	105.83 (12)
C7—O2—Li1^ii^	146.29 (11)	O3—Li1—O1^i^	112.89 (12)
C3—N2—N1	118.83 (12)	O2^iii^—Li1—O1	119.50 (12)
O2—C7—O1	125.47 (12)	O3—Li1—O1	111.69 (13)
O2—C7—C4	116.99 (11)	O1^i^—Li1—O1	90.67 (10)
O1—C7—C4	117.54 (11)	O2^iii^—Li1—Li1^i^	123.03 (16)
N1—C6—C5	123.32 (13)	O3—Li1—Li1^i^	122.65 (15)
N1—C6—H6	118.3	O1^i^—Li1—Li1^i^	45.65 (7)
C5—C6—H6	118.3	O1—Li1—Li1^i^	45.02 (7)
C6—N1—N2	119.95 (12)	Li1—O3—H31	133.5 (19)
N2—C3—C4	123.52 (13)	Li1—O3—H32	118.7 (18)
N2—C3—H3	118.2	H31—O3—H32	104 (3)

Symmetry codes: (i) −*x*, −*y*+1, −*z*; (ii) *x*, −*y*+1/2, *z*+1/2; (iii) *x*, −*y*+1/2, *z*−1/2.

### Hydrogen-bond geometry (Å, °)

**Table:**

*D*—H···*A*	*D*—H	H···*A*	*D*···*A*	*D*—H···*A*
O3—H32···N1^iv^	0.86 (3)	1.93 (3)	2.7910 (18)	175 (3)
O3—H31···N2^v^	0.85 (3)	2.33 (3)	3.1272 (19)	155 (2)

Symmetry codes: (iv) *x*−1, *y*, *z*−1; (v) *x*−1, −*y*+1/2, *z*−1/2.
